# HIV-1 Tat alters neuronal intrinsic excitability

**DOI:** 10.1186/s13104-018-3376-8

**Published:** 2018-05-04

**Authors:** Walter Francesconi, Fulvia Berton, Maria Cecilia G. Marcondes

**Affiliations:** 1 San Diego Biomedical Research Institute, 10865 Road to the Cure, San Diego, CA 92121 USA; 2 Department of Anatomy and Cell Biology, College of Medicine, University of Illinois Chicago, 6068 COMRB MC 512, Chicago, IL 60612 USA; 3 The Scripps Research Institute, 10550 North Torrey Pines Rd., La Jolla, CA 92037 USA

**Keywords:** neuroHIV, HIV Tat, Hippocampal neurons, Electrophysiology, Synaptic transmission

## Abstract

**Objective:**

In HIV+ individuals, the virus enters the central nervous system and invades innate immune cells, producing important changes that result in neurological deficits. We aimed to determine whether HIV plays a direct role in neuronal excitability. Of the HIV peptides, Tat is secreted and acts in other cells. In order to examine whether the HIV Tat can modify neuronal excitability, we exposed primary murine hippocampal neurons to that peptide, and tested its effects on the intrinsic membrane properties, 4 and 24 h after exposure.

**Results:**

The exposure of hippocampal pyramidal neurons to Tat for 4 h did not alter intrinsic membrane properties. However, we found a strong increase in intrinsic excitability, characterized by increase of the slope (Gain) of the input–output function, in cells treated with Tat for 24 h. Nevertheless, Tat treatment for 24 h did not alter the resting membrane potential, input resistance, rheobase and action potential threshold. Thus, neuronal adaptability to Tat exposure for 24 h is not applicable to basic neuronal properties. A restricted but significant effect on coupling the inputs to the outputs may have implications to our knowledge of Tat biophysical firing capability, and its involvement in neuronal hyperexcitability in neuroHIV.

## Introduction

Neuronal plasticity and regeneration are interrelated phenomena, tightly dependent on the normal functioning of the Central Nervous System (CNS). Changes in glia drastically affect neuronal capacity to recover and rewire [1–3]. Microglia invade the CNS at prenatal stages, increasing density during the first weeks of postnatal life, reaching a maximum by P18, concomitant to an intense synaptogenesis period [4–6]. Microglia express cytokines, neurotrophins [7, 8], glutamate and NO [9–12], which regulate synaptic properties. Importantly, microglia are critical during Human Immunodeficiency Virus (HIV) infection, because they get infected by virus carried into the brain by macrophages at early time points [13, 14].

The consequences of HIV in the brain include high incidence of neurological dysfunctions, even in the post-antiretroviral age [14]. Of all HIV-1 peptides, Tat is involved in viral transcription, and is unconventionally secreted by infected targets [15–18], with consequences to neighboring cells, including neurons. HIV-Tat has been linked to impaired learning and memory, and gray matter deficits [19, 20], suggesting its involvement in the development of HIV-associated neurological disorders (HAND).

The hippocampus is a brain region involved in cognition, and memory formation, organization, and retrieval, where the main cell type is the excitatory glutamatergic pyramidal neuron, integrating spatial, contextual, and emotional information, while transmitting all outputs to cellular targets throughout the brain, in response to glutamate, a key neurotransmitter [21]. Pyramidal cells in the CA1 and subiculum regions carry output by firing individual or high frequency bursts of action potentials (AP), increasing synaptic communication through evoking a postsynaptic spike [22, 23]. They also participate in plasticity and place field development [24, 25]. Excitatory synaptic transmission in hippocampal neurons is susceptible to changes, contributing to cognitive impairments. Due to the abundance of these neurons, the hippocampus is crucial for recalling when and where an event occurred, or ‘episodic memory’ [26], one of the first functions lost in HAND and in aging [27, 28]. Importantly, we have demonstrated that HIV Tat prevents long-term potentiation in the hippocampal CA1 region [29]. Our goal was to further characterize the neuronal response to HIV Tat in primary cultures.

Neuronal membrane properties are characterized by means of the input–output response function, giving the rate of AP discharge as a function of the injected current strength. The linear relationship between neuronal input and output is defined by the rheobase (minimum synaptic input that generates an AP), and by the slope (gain). The gain control is a central feature of neural information processing [30]. Changes in gain control, associated with alterations in the conductance of voltage-gated channels such as the A-type (IA), the delayed-rectifier K+ (Id) and L-type voltage-gated Ca^2+^ (IKCa) channels, are critical in several pathophysiological conditions. An increase in IA, Id and IKCa reduces neuronal gain. In contrast, increases in the slow voltage-gated Ca^2+^ channel conductance (GCaS) increase neuronal gain. The hyperpolarization-activated inward channel (Ih) is ‘gain neutral’. The functional relevance of such changes include protecting neurons from over-excitation during ischemia, infection, or aging, and for making neurons more excitable during associative learning [31–36].

We used the slope of the fitted linear function (gain, I–O slope) as a quantitative measure of biophysical firing capabilities under DC step stimulation [37], to examine whether HIV-1 Tat can modulate hippocampal neuron properties, explaining changes in memory functions experienced by HIV+ subjects. In neuronal primary cultures we modeled Tat exposure, and tested its effects on excitability.

In cell line studies, Tat internalization by neurons was detected at 4 h [38]. Effects on molecular functions and morphology were detected at 24 h [39, 40], preceding neurotoxicity at 48 h [38]. The ability of Tat to modify neuronal excitability in the primary hippocampal neuron culture system was tested on whole cell patch electrophysiological testing paradigms, at 4 and 24-h time points. We found a significant effect of Tat at 24 h after exposure.

## Main text

### Methods

#### Hippocampal cultures

Animal use was approved by Institutional Animal Care and Use Committees of The Scripps Research Institute (TSRI) and San Diego Biomedical Research Institute. In three independent experiments, two pregnant C57Bl/6 females, 5–8 weeks old, were purchased from TSRI Department of Animal Resources. E17 pups [41] (~ 7/experiment) were sacrificed by CO_2_ inhalation. Hippocampi were dissected in Ca^2+^/Mg^2+^-free, HEPES-buffered Hank’s balanced salt solution (HBSS), pH7.45, dissociated through flame-narrowed Pasteur pipettes of decreasing aperture, and resuspended in DMEM without glutamine, 10% fetal bovine serum and penicillin/streptomycin (100 U/ml and 100 μg/ml, respectively). Cells were plated (120,000/dish) onto 25 mm round Matrigel (200 μl, 0.2 mg/ml; BD Biosciences) pre-coated cover glass glued to cover a 19-mm-diameter opening drilled through the bottom of a 35 mm Petri dish. Neurons were grown in 10% CO_2_, at 37 °C, and fed on days 1 and 6, by exchanging 75% of the media with DMEM containing 10% horse serum and penicillin/streptomycin. In all experiments, pyramidal-shaped neurons behaved as such in patch-clamp.

#### Tat stimulation

Recombinant HIV-1 Tat (Clade B) was from the National Institutes of Health AIDS Research and Reference Reagent Program, Division of AIDS, National Institute of Allergy and Infectious Diseases. In control experiments, Tat was heat-inactivated at 85 °C for 30 min. The cells were incubated with Tat 10 ng/ml (6.4 nM). Tat (or inactivated Tat) was added to the media 4 or 24 h prior to recordings. Coded cultures were removed from 37 °C, and a 95% O_2_/5% CO_2_ injector was placed in wells, under a differential interference contrast microscope (Leica). Electrophysiology was performed in randomized cultures, in a blinded manner.

#### Whole-cell patch clamp recordings and intracellular stimulation

Patch clamp recordings and intracellular stimulation were performed and captured using Multiclamp 700B amplifier (Axon Instruments). Stimulus waveforms were generated using data acquisition software DASYLab11.0 (National Instruments) in Windows computer equipped with National Instruments PCI-MIO-16-E4 board. We used rectangular hyperpolarizing and depolarizing current pulses as stimuli for physiological characterization. Specifically, 350 ms current pulses starting from − 200 pA, were incremented by 10 pA. Voltage responses were analyzed using software developed by Delphi, 2009, using the following parameters: Resting membrane potential, Resting input resistance, Rheobase, AP Threshold, AP amplitude and duration, AP after hyperpolarization, and the I-O function (gain). The spontaneous excitatory postsynaptic currents (sEPSCs) were recorded in voltage clamp mode at − 70 mV holding potential. The spontaneous inhibitory postsynaptic current (sIPSC) was recorded in voltage clamp mode at − 40 mV holding potential.

#### Statistical analysis

Using Prism5 (GraphPad Software Inc, La Jolla, CA), we examined deviations from normality in the data using Kolmogorov–Smirnov test. Slopes were tested using Pearson’s correlation coefficient (r) (p < 0.01), and linear regression. The mean of input–output relationship [42] was compared by ANOVA, and Bonferroni’s post hoc test (p < 0.05). Individual parameters between controls and Tat 24 h were compared using Student’s t test.

### Results

We examined whether dysfunctional properties are detectable in hippocampal neurons exposed to HIV-1 Tat for 4 and 24 h. We measured electrophysiological properties in the pyramidal subset, in comparison to cultures treated with inactivated Tat. In both time-points, control cultures showed similar behaviors and thus measurements were pooled.

**Fig. 1:**
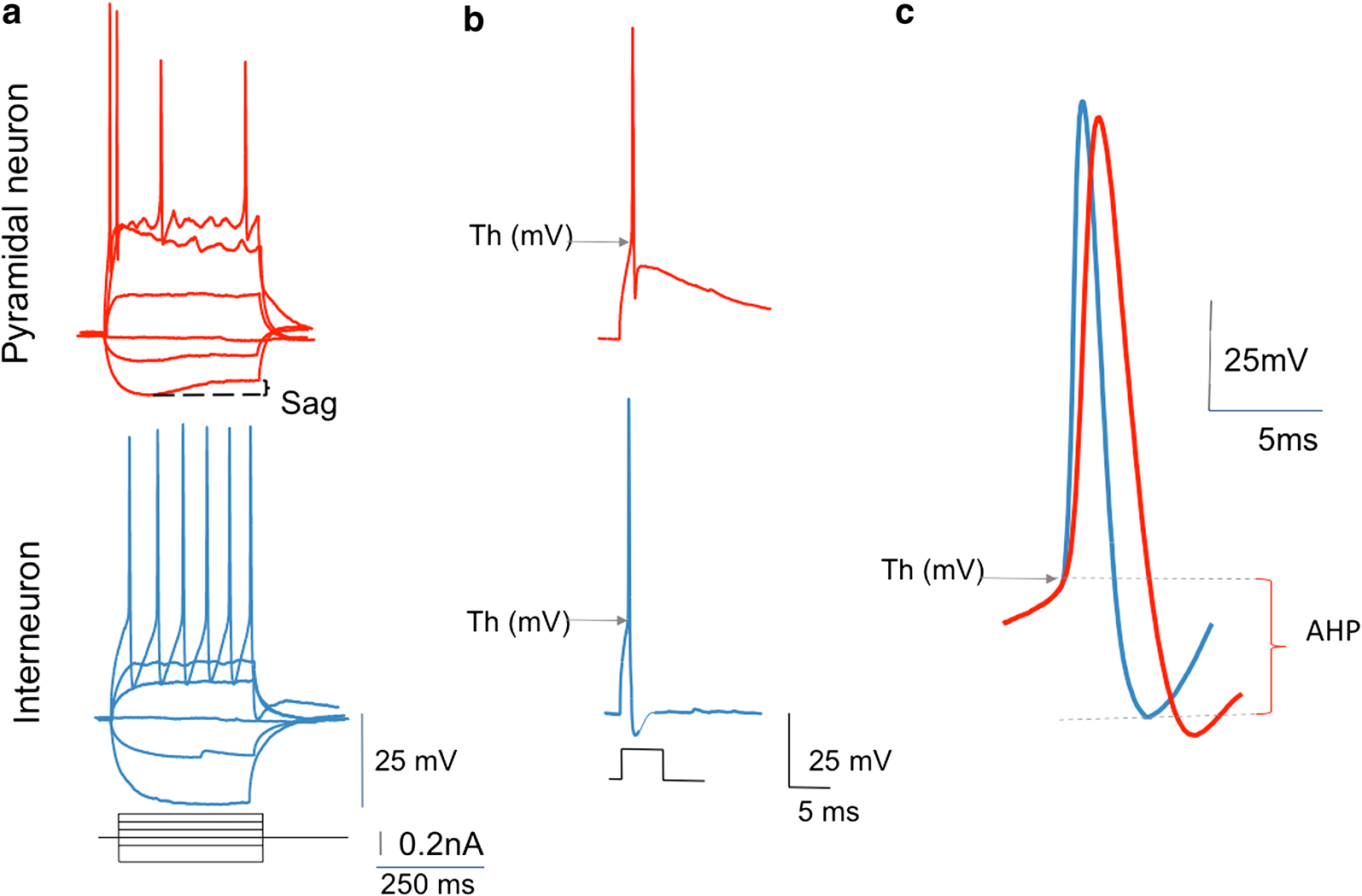
Summary of baseline electrophysiological function behaviors that distinguish neuronal subsets in hippocampal primary cultures, and that were utilized to examine the effect of Tat exposure. The expected recording profiles distinguish pyramidal glutamatergic neurons (red lines) from GABAergic interneurons (blue lines), as seen in this representative electrophysiological characterization of hippocampal neurons in culture. **a** Whole cell patch-clamp recording from pyramidal neuron (upper red traces) and interneuron (lower blue traces). Voltage responses to hyperpolarizing and depolarizing current pulses differ in the 2 types of neurons. Pyramidal neurons show their characteristic voltage sag during hyperpolarizing pulse, and a strong adaptation. In contrast, interneurons fire at higher frequency without signs of adaptation. **b** Action potentials (AP) evoked by a short depolarizing current pulse. In the pyramidal neurons, AP shows a depolarizing potential during the repolarization phase (upper trace). In interneurons, the AP is followed by a fast hyperpolarization, referred to as fast after-hyperpolarization (fAHP) (lower trace), which is a characteristic oh hippocampal interneurons. **c** The action potential width is shorter in interneurons (blue lines) when compared to pyramidal neurons (res lines). We tested the hypothesis that HIV Tat has the ability to interact with neurons affecting their performance, which can be detectable by changes (increase or decrease) in the pulse current intensity that is necessary to elicit an AP

Figure 1 summarizes the parameters characterizing neuronal subsets. Baseline recordings showed that hippocampal neuronal subsets in primary cultures exhibit expected behaviors, and are a valid system for studying the direct effects of HIV peptides such as Tat, or neuroimmune factors. In control cultures, pyramidal Glutamatergic neurons (Fig. 1, red lines) differed from GABAergic interneurons (Fig. 1, blue lines), in recording patterns during whole cell patch-clamp assays. Pyramidal neurons displayed characteristic voltage sag during hyperpolarizing pulse, and strong adaptation (Fig. 1a, upper trace), while interneurons fired at higher frequency without signs of adaptation (Fig. 1a, lower trace). Regarding AP evoked by a short depolarizing current pulse, pyramidal neurons showed depolarizing potential during the repolarization phase (Fig. 1b, upper trace), while in interneurons, AP was followed by a fast after-hyperpolarization (fAHP) (Fig. 1b, lower trace). The AP width was shorter in interneurons (Fig. 1c, blue line) compared to pyramidal neurons (Fig. 1c, red line). All parameters were according to predicted results for these subsets.

**Fig. 2:**
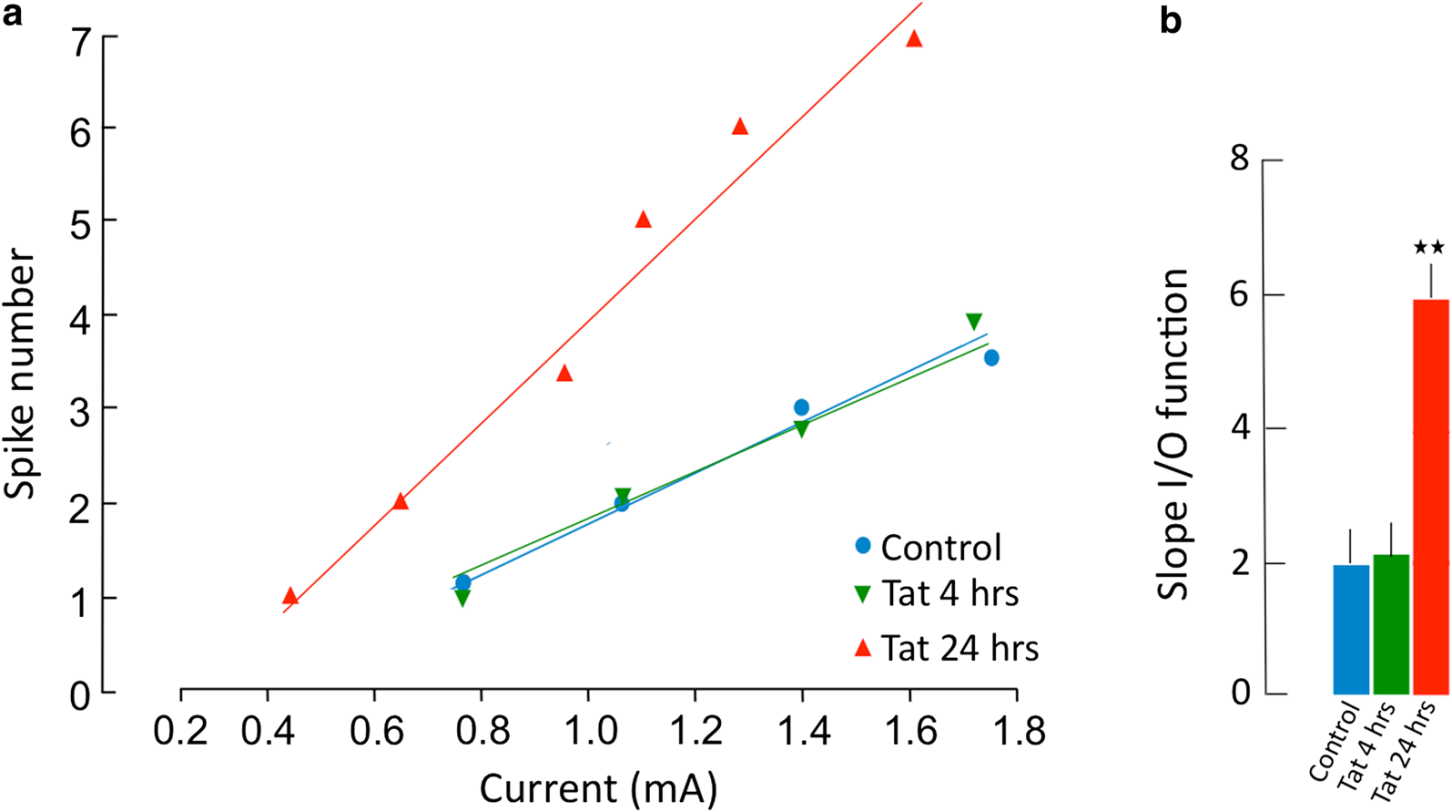
Input/output function slope (gain) is enhanced in Tat-treated pyramidal neurons. **a** The graph shows the mean number of action potentials (AP) generated by the pyramidal neurons is response to depolarizing current pulses of different intensities, in one representative experiment. In a defined range of current intensities, the relationship between number of spikes and current intensity is linear. The slope of this function, referred to as “Gain”, is higher in the pyramidal neurons treated wit Tat for 24 h (red), when compared to the control (blue) or to Tat for 4 h (green) in pyramidal neurons in culture, as determined by Pearson’s correlation coefficient. **b** The bar graph shows the mean (± SEM) slope of the input/output (I/O) function of controls (n = 10, blue bar) and Tat treated neurons for 4 h (n = 5, green bar) or for 24 h (n = 5, red bar). The mean slope of the I/O function was 1.95 ± 0.53 (SEM) for untreated pyramidal neurons and 5.89 ± 0.59 (SEM) for Tat treated neurons (p < 0.002, ANOVA followed by Bonferroni’s test)

Using this system, we tested the hypothesis that Tat can directly affect neuronal performance, detectable by increase or decrease in pulse current intensity necessary to elicit an AP. We have focused on glutamatergic pyramidal neurons, due to their excitatory role in circuitry architecture, and due to their consistent pattern of Tat-elicited changes, compared to control neurons. All cultures showed a linear relationship between the injected current and the number of spikes, determined by Pearson’s coefficient (Control r^2^ = 0.9969, p = 0.0031; Tat 4 h r^2^ = 0.9989, p = 0.0011; Tat 24 h r^2^ = 0.9827, p = 0.0004). Compared to control cultures, Tat did not alter neuronal behaviors at 4 h. However, a strong increase of the gain function was seen at 24 h (Fig. 2). The slope comparison by linear regression revealed a significant difference, with F = 16.4446, DFn = 2, DFd = 8, and p = 0.001465, and with a 0.15% chance of randomly choosing data points exhibiting these differences. The comparison of the mean of the slopes input–output (I/O) relationship within the tested interval showed a significant difference between Control and Tat 24 h (p = 0.002, Bonferroni’s p < 0.05). Yet, pyramidal neurons treated with Tat did not alter the RMP, input resistance, rheobase (or the minimal depolarizing current input that generates an AP), and AP threshold at 24 h (Table 1).

**Table 1:** Effect of HIV-1 Tat peptide on resting membrane potential (RMP), input resistance (Rin), action potential threshold, and rheobase in hippocampal pyramidal neurons

	RMP (mV)	Rin (MW)	Threshold (mV)	Rheobase (nA)	Gain (Spike/_S_)
Control	68.52 ± 0.33	53.06 ± 0.889	43.36 ± 0.201	0.33 ± 0.071	1.9 ± 0.7
HIV-1 Tat 24 h	68.56 ± 0.63	53.04 ± 1.12	43.7 ± 0.466	0.31 ± 0.032	5.9 ± 0.6
t test	0.956	0.989	0.522	0.543	1.678E−10**

The Tat peptide was added to the cultures, and electrophysiological parameters were tested at 24 h

### Discussion

We found that Tat critically affects neuronal intrinsic excitability in cultured hippocampal pyramidal neurons, as a result of a lower reactive threshold to current. This effect was not observed at 4 h after HIV-1 Tat exposure, but only at the 24 h time point.

In cell line studies, conflicting results relate to the diversity of models. In a neuro-epithelial-like stem (NES) cell line from human fetal hindbrain, Tat at 10 times lower doses than in our study caused deep changes in gene expression and cytoskeletal structure at 24 h, and a reduction of output excitability at 48 h [40], likely due to neurotoxicity.

In primary cultures, Tat-induced changes are subset, dose and time-depend. For instance, in rat dorsal root ganglion small diameter capsaicin-sensory neurons, Tat at 20 nM greatly enhanced excitability, suggesting a direct role in pain [43]. On the other hand, studies in rat hippocampal neurons show Tat-induced biphasic changes in NMDA-evoked increases in intracellular Ca^2+^, with consequences to spontaneous activity [42, 44]. Tat (at 5-fold higher concentrations than in our study) acutely reduced spontaneous spike frequencies while increasing AP bursts amplitude and duration, followed by attenuation, and adaptation at 24 h. These changes were hypothesized to result from aberrant network activity, attributed to changes in NMDA-gated intracellular Ca^2+^, mediated by Src kinase and NO signaling [42, 45]. Our results complement those, suggesting that neuronal adaptability to lower Tat concentrations may be relative, or not applicable to all aspects of neuronal function. Our findings are in agreement with the excitatory effect of Tat on cultured human fetal neurons, and rat hippocampal slices [46–48].

The neuronal ability to receive and transmit information depends on neurotransmitter concentrations in presynaptic terminals, numbers and intrinsic properties of postsynaptic receptors on dendritic trees, and receiving synaptic inputs, which depend on the type of voltage-dependent membrane ionic channels. These channels, upon inputs and AP, allow ionic movement, changing the excitability. We observed that Tat increases neuronal excitability, or the slope of the input–output relationship. Tat may enhance firing via Ca^2+^ influx [49, 50], by prolonging Ca^2+^ potentials mediated by L-channels. Importantly, neuronal voltage-gated K+ channels (Kv) are involved in memory processes [51, 52], and in acquired neuronal channelopathies observed in HIV-associated neurocognitive disorders [53]. Further studies must determine what conductance is affected by Tat exposure, and whether these findings apply to neuroHIV models in vivo. If so, these may have consequences to how HIV in the brain affects perception, reactivity to sensory stimulation, and memory, in part explaining HIV-associated neurobehavioral changes.

### Conclusions

HIV-Tat acts on hippocampal pyramidal neurons by lowering the current pulse intensity threshold that elicits an action potential response, and increases gain slope 24 h following exposure, indicating an enhanced intrinsic neuronal excitability.

## Limitations

This study was in isolated neurons in culture, not subjected to neuroimmune changes, or active infection, differing from the HIV-infected brain. Yet, it is a system for the examination of direct effects of HIV and its peptides, and that can accommodate complexities from neuroimmune cells. More studies are need for identifying mechanisms by which Tat affects excitability, without affecting other functions.

## Abbreviations

Tat: trans-activator of Transcription
CNS: central Nervous System
P18: pre-natal day 18
NO: nitric oxide
HIV: human immunodeficiency virus
HAND: HIV-associated neurocognitive disorders
CA1: cornu ammonis layer 1
AP: action potential
RPM: resting membrane potential
IA: A-type voltage gated channel
Id: delayed rectifier K+ gated channel
IkCa: L-type voltage-gated Ca^2+^ channel
GCaS: voltage-gated Ca^2+^ channel conductance
Ih: inward channel
I-O: input: output
DC: direct current
Hr: hour
Hrs: hours
E17: embryonic day 17
HEPES: 4-(2-hydroxyethyl)-1-piperazineethanesulfonic acid
HBSS: Hank’s balanced salt solution
SDBRI: San Diego Biomedical Research Institute
TSRI: The Scripps Research Institute
DMEM: Dulbecco’s modified Eagle’s medium
AIDS: acquired immunodeficiency syndrome
pA: pico-amperes
mS: millisecond
sIPSC: inhibitory post synaptic current
mV: millivolts
ANOVA: analysis of variance
fAHP: fast after hyperpolarization
NES: neuro-epithelial-like stem cell
NMDA: *N*-methyl-d-aspartate
Kv: voltage-gated K+ channels
